# Toward a comprehensive drug ontology: extraction of drug-indication relations from diverse information sources

**DOI:** 10.1186/s13326-016-0110-0

**Published:** 2017-01-10

**Authors:** Mark E Sharp

**Affiliations:** Scientific Information Management, Merck Research Laboratories, 770 Sumneytown Pike, West Point, Philadelphia, PA 19486 USA

**Keywords:** Drug indications, Drug-disease relations, Drug ontologies, Drug information integration, UMLS, WHO-ATC

## Abstract

**Background:**

Drug ontologies could help pharmaceutical researchers overcome information overload and speed the pace of drug discovery, thus benefiting the industry and patients alike. Drug-disease relations, specifically drug-indication relations, are a prime candidate for representation in ontologies. There is a wealth of available drug-indication information, but structuring and integrating it is challenging.

**Results:**

We created a drug-indication database (DID) of data from 12 openly available, commercially available, and proprietary information sources, integrated by terminological normalization to UMLS and other authorities. Across sources, there are 29,964 unique raw drug/chemical names, 10,938 unique raw indication ”target” terms, and 192,008 unique raw drug-indication pairs. Drug/chemical name normalization to CAS numbers or UMLS concepts reduced the unique name count to 91 or 85% of the raw count, respectively, 84% if combined. Indication ”target” normalization to UMLS ”phenotypic-type” concepts reduced the unique term count to 57% of the raw count. The 12 sources of raw data varied widely in coverage (numbers of unique drug/chemical and indication concepts and relations) generally consistent with the idiosyncrasies of each source, but had strikingly little overlap, suggesting that we successfully achieved source/raw data diversity.

**Conclusions:**

The DID is a database of structured drug-indication relations intended to facilitate building practical, comprehensive, integrated drug ontologies. The DID itself is not an ontology, but could be converted to one more easily than the contributing raw data. Our methodology could be adapted to the creation of other structured drug-disease databases such as for contraindications, precautions, warnings, and side effects.

**Electronic supplementary material:**

The online version of this article (doi:10.1186/s13326-016-0110-0) contains supplementary material, which is available to authorized users.

## Background

Biomedical information overload and the potential of formal ontologies to help overcome it are well recognized [1–3]. Information overload is but one threat to the viability of the traditional pharmaceutical industry. Others include the rising costs of laboratory research, clinical trials, litigation over anomalous harmful side effects, and increasing times to market [4]. The success of the Gene Ontology (GO) as an *in silico* molecular biology research tool [5] suggests that drug ontologies could have a similar impact on drug research. The advance of practical ontologies into the pharmaceutical domain has been much anticipated [6–8], and is becoming evident [9, 10].

Pioneering reports on ontology-based, *in silico* drug discovery have emerged [11–13]. The basic goal is ontology-assisted inference of surprising and/or more-likely-to-succeed new drug candidate compounds for known uses, thus cutting costs and time to market. Drug ontology-assisted inference could also be applied to finding new uses for known compounds (drug repurposing) [14], or “personalized” genome-dependent safety/efficacy profiling (pharmacogenomics) [15–18]. These ontologies include drug relations to chemically similar compounds, diseases (therapeutic classifications, indications, side effects), and biological pathways (mechanisms of action, molecular target proteins or their genes, secondary disease-gene and protein-protein interactions). In principle, such ontologies could be expanded to encompass many more dimensions of drug information [19, 20]; that is, they can be made more comprehensive.

For further progress in building comprehensive drug ontologies, rich and well-structured knowledge (content) about biological pathways and chemically similar compounds is readily available from resources such as GO, GenBank [21], DrugBank [22], PubChem [23], and ChemIDplus [24]. Rich drug-disease knowledge also is readily available, but usually as unstructured (“free”) text; e.g., DailyMed [25]. Thus the well-structured but relatively shallow WHO-ATC drug classification [26] has been utilized as a source for drug-disease knowledge [12, 13].

It is important to distinguish between diseases, indications, contraindications, side effects, and other such dimensions of drug information. A drug indication can be a disease^1^ that the drug is “used for” (i.e., to treat, prevent, manage, diagnose, etc.). An important subset are *approved* indications which have been through a formal, country-specific regulatory vetting process. But drugs can also be indicated for medical conditions which may not be considered diseases, such as pregnancy. Drugs can also be indicated for procedures, such as contrast media for radiology. In ontological terms, medical conditions (of which diseases are a subclass) and medical procedures constitute the *range* of drug indications. They also constitute the range of very different, even orthogonal, drug relations such as contraindications, precautions, and warnings. The range for side effects, on the other hand, is arguably limited to diseases. Thus it is important to specify which of these relations is being addressed. This paper addresses indications, but much of it is extensible to other drug-disease relations.

## Methods

We created a drug-indication database (DID) using content from openly available, commercially available, and Merck proprietary information resources. To integrate the data, we attempted to identify distinct “triples” of a drug, indication, and indication subtype (treat, prevent, manage, diagnose, etc.), and then normalize each component to a standard terminology or code. The raw data varied widely in format, from well-structured, vocabulary-controlled triples to hierarchical classifications to free text. While the DID itself is not an ontology, it could be converted to one more easily than the contributing raw data.

### Sources

Raw data on drug/chemical-indication relations were collected from the following resources.

### DailyMed

DailyMed [25] is a free drug information resource provided by the U.S. National Library of Medicine (NLM) that consists of digitized versions of drug labels (also called “package inserts”) as submitted to the U.S. Food and Drug Administration (FDA). The information format of the labels is mostly free text but with standard section headings, including “Indications & Usage.” DailyMed was of special interest because of its comprehensive coverage, open availability, and the package inserts’ combination of format consistency, rich detail, and provenance (manufacturer-written, scientifically vetted, and FDA-approved).

### DrugBank

DrugBank [22] “is a unique bioinformatics and cheminformatics resource that combines detailed drug (i.e. chemical, pharmacological and pharmaceutical) data with comprehensive drug target (i.e. sequence, structure, and pathway) information” provided by the University of Alberta. Many records include an explicit *Indication* field populated with free text values, and leveraging these was of special interest due to DrugBank’s rich coverage of molecular target information.

### MeSH PA

MeSH (Medical Subject Headings) [27] is NLM’s controlled vocabulary used to index Medline/Pubmed [28] articles by scientific topics including drugs, chemicals, diseases, and other biomedical conditions, processes, and procedures. MeSH has ontology-like hierarchical and other relationships between concepts, but it does not consistently link drugs to diseases/conditions/processes explicitly (e.g., “Aspirin” to “Fever”). It does, however, have a special *Pharmacological Action* (PA) relationship which links drugs and other chemicals to therapeutic classes (e.g., “Aspirin” to “Antipyretics”) which could be mapped to diseases/conditions/processes (e.g., “Antipyretics” to “Fever”).

### NDFRT

NDFRT (National Drug File Reference Terminology) [29] is produced by the U.S. Veterans Health Administration and is openly available from several resources including NLM’s UMLS (Unified Medical Language System) [30]. Like MeSH PA, NDFRT consists of controlled vocabulary terms connected by specific relationship names, five of which could be considered pointers to indications or PA’s: *may_treat, may_prevent, may_diagnose, has_mechanism_of_action, has_physiological_effect*.

### PDR

PDR (Physicians’ Desk Reference) is “a commercially published compilation of manufacturers’ prescribing information (package insert) on prescription drugs, updated annually.” [31] Its long history (65 editions) and ubiquitous hardcopy availability give PDR a certain provenance. “Section 3 - Product Category Index” classifies drugs (trade names) by disease (e.g., “ALCOHOL DEPENDENCE”) and/or PA (e.g., “ANALGESICS”).

### ChEBI

ChEBI (Chemical Entities of Biological Interest) [32] consists of a database and ontology supplied by the European Bioinformatics Institute. The *has_role* relationship of the ontology connects drugs and chemicals to functions, including PAs (e.g., “antibacterial drug”; “anti-ulcer drug”; “proton pump inhibitor”).

### CTD

CTD (Comparative Toxicogenomics Database) [33, 34] is supplied by North Carolina State University and Mount Desert Island Biological Laboratory, Salisbury Cove, Maine. The *Chemical-Disease Associations* file consists of pairs of MeSH terms connected by the relationships *therapeutic* and/or *marker/mechanism* and annotated by evidence type; we used the “direct evidence” subset.

### USAN TC

**Fig. 1:**
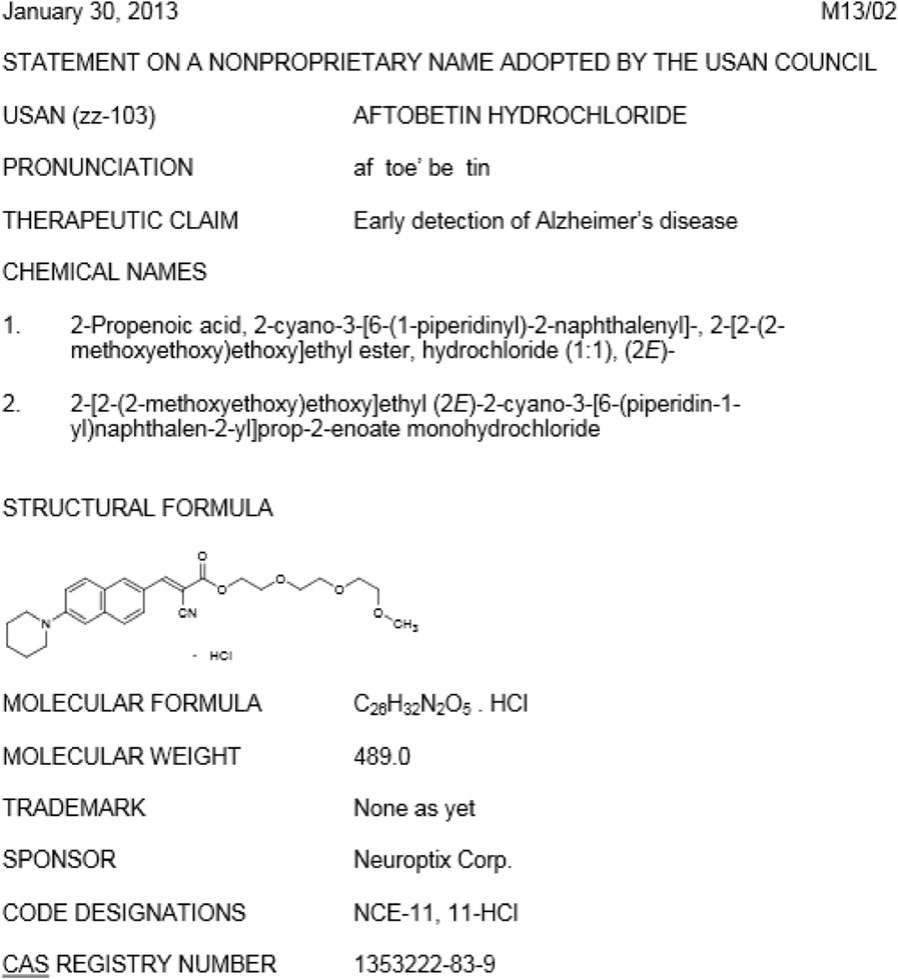
Example USAN raw data

USANs (United States Adopted Names) are the official U.S. generic names chosen for drugs by the USAN Council in consultation with the drug’s sponsoring company [35]. Each name has a variety of structured (but not necessarily vocabulary controlled) relations signifying proprietary, chemical, and therapeutic information. This information is published annually in the *USP Dictionary of United States Adopted Names (USAN) and International Drug Names* [36] and monthly by the American Medical Association (AMA) [37] (Fig. 1), and Merck encodes it in our internal vocabulary system (“eVOC”). The Therapeutic Claim (TC) values include disease names, PAs, and indication subtypes such as “treatment of” and “prevention of.”

### WHO-ATC resources

WHO-ATC (World Health Organization Anatomic-Therapeutic-Chemical) is a five-level drug classification hierarchy specifying (typically, from top to bottom) the anatomical system acted upon, therapeutic action, and chemical nature of the drug. The hierarchy can convey multiple indications/PAs for a given drug. WHO-ATC is widely accepted as a standard for drug classification, including in the Merck eVOC system. We obtained WHO-ATC data from two WHO datasets purchased by Merck and additional mappings in eVOC; these are referred to as WHO_ATC [38], WHO_DD [39], evoc_ATC, and evoc_eProj in the rest of this document. (All evoc_eProj and some evoc_ATC data represent Merck proprietary information and therefore have been removed from the attached DID subset, Additional file 1.)

### Parsing and filtering

These resources and their contributions to our database are summarized in Table 1. “Parsed” refers to converting the raw data to triples of a drug, indication, and indication subtype. In the process of parsing, some raw data was found to be irrelevant, redundant, and/or intractable, and therefore was removed from further processing (“filtered”). Differences in contribution counts from “filtered” to “parsed” correlate inversely with how well-structured and vocabulary-controlled were the raw source data, from low (ChEBI, CTD, MeSH PA, NDFRT) to high (DailyMed, DrugBank).

**Table 1:** Source contributions of drug-indication data

source abbrev	source name or description	subset if any	version/date	number of drug-indication pairs		
				initial	filtered	parsed
ChEBI	Chemicals of Biological Interest Ontology	has_role relations	104/June 1, 2013	16,415	8,598	8,598
CTD	Comparative Toxicogenomics Database	Chemicals-Diseases Associations,“direct evidence” subset	May 2, 2014	82,000	81,214	81,214
DailyMed	NLM’s database of FDA package inserts	single component title (product name) & Indications sections with tractable text length (<540)	March 20, 2011	15,834	1,612	3,840
DrugBank	U. Alberta open access DB of drug target and other info	title (drug name) & Indications sections	3.0/2011	1,599	1,595	6,004
MeSH PA	Medical Subject Headings Pharmacologic Action relations		2013/Dec. 3, 2012	26,293	25,847	25,908
NDFRT	National Drug Formulary Reference Terminology	may_treat & may_prevent relations	2009AA (UMLS)	50,775	5,294	5,294
PDR	Physicians’ Desk Reference	Section 3 - Product Category Index	2006	3,150	1,204	2,169
USAN_TC	United States Adopted Names Therapeutic Claims		March 31, 2014 (eVOC)	6,569	5,954	7,234
WHO_ATC	World Health Organization Anatomic-Therapeutic-Chemical, Defined Daily Dose index		2005	16,276	7,807	9,004
WHO_DD	World Health Organization Drug Dictionary	single generic compounds with ATC codes (minus 2005 WHO-ATC overlap and herbals BNA = “9…”)	Sept. 2013	40,736	21,764	25,674
evoc_ATC	WHO-ATC codes in Merck’s eVOC *generic names* dictionary	single generic compounds with ATC codes (minus WHO-ATC & WHO-DD overlap)	May 6, 2014	65,552	16,269	19,093

The numbers refer to candidate drug-indication pairs in the initial raw data extract (initial), after filtering for internal redundancy, relevance, and/or tractability (filtered), and after parsing of free text into single concepts (parsed) as described in the main text. The “filtered” count is the number of unique pairs of raw drug name (DID column D) and indication “entire value/string” (column AQ), while the “parsed” count is the number of unique pairs of raw drug name and indication “target/substring” (column AR). evoc_eProj data are not shown

Filtering is not qualitatively different from initial subsetting (Table 1, column 3). For example, ChEBI’s, CTD’s, and MeSH PA’s relatively large initial contributions can be attributed to their higher coverage of non-drug chemicals and non-therapeutic quasi-indications (e.g., “Carcinogens”; “Mutagens”). These could be considered irrelevant to pharmacy/prescription applications of the DID, but were left in for drug discovery applications. ChEBI’s contribution was reduced 48% by filtering out irrelevant (non-indication) *has_role* objects (e.g., “metabolite”; “prodrug”; “epitope”), but CTD’s “marker/mechanism” subset (63%) was not removed due to its potential use in future analysis. DailyMed’s filtering reduction was even larger but aimed at very different targets: combination products (20%), intractably long (>539 characters) “Indication & Usage” texts (37%), and redundant “Indication & Usage” values paired with the same drug generic name differing only by dosage, formulation, trade name, or supplier (33%). NDFRT’s (90%) and PDR’s (62%) filtering reductions were also due primarily to conflating various forms (trade names, in PDR’s case) of the same generic name.^2^

Initial counts from the WHO-ATC resources are based on viewing each level of the WHO-ATC hierarchy as a separate indication, rather than combining them into a single raw term. Filtering resulted in reductions of 52% (WHO_ATC), 47% (WHO_DD), and 75% (evoc_ATC) reflecting removal of combination and ill-formed drug names, and non-indication and redundant classification terms (e.g., “Antithrombotic Agents” at nested hierarchical levels [B01 and B01A]).

It must be emphasized that the parsing, filtering, and normalizing (see below) done in this work employed a wide variety of ad hoc methods and manual curation commensurate with the raw data/source diversity.

### Normalizing drug names

Various types of drug identifiers are exemplified in Fig. 1, including a *generic name* (in this case a USAN, “aftobetin hydrochloride”), *chemical names*, a *structural formula*, sponsor *code designations*, and a *CAS* (Chemical Abstracts Service) *Registry Number*. Other types not shown in Fig. 1 include *trade names* (e.g., “Tylenol” corresponding to the generic name “acetaminophen”), FDA’s *UNII* (Unique Ingredient Identifier; e.g., “A1FCZ940WA” for “aftobetin hydrochloride”), and *InChI* (International Chemical Identifier) *Key* (e.g., “GMWHTUNMFTUKHH-NDUABGMUSA-N” for “aftobetin hydrochloride”) [40]. The equivalence of such terms for the exact same chemical entity can sometimes be debated due to details such as isomerism, salt forms, hydration, formulation, and dosage, but they are commonly considered synonyms, with the generic name as the preferred term (PT).

Thus, to parse out the drug identifier in each raw drug-indication record, we looked for source database fields or elements containing these types of terms, and attempted to normalize them to generic names using the sources’ own and/or other synonym dictionaries. These dictionaries included those available from ChemIDplus, ChEBI, CTD, DrugBank, UMLS, and Merck’s eVOC. In addition, to resolve conflicts among these dictionaries, we attempted to derive a “preferred PT” via CAS number mapping and ranking the dictionaries in the order ChemIDplus > ChEBI > DrugBank > eVOC > CTD. For example, in ChemIDplus the PT for CAS number 103-90-2 is “acetaminophen” but in ChEBI it is “paracetamol.” Thus the DID enables ChEBI drug-indication data for “paracetamol” to be grouped with other sources’ drug-indication data for “acetaminophen.” UMLS is not a rich source of CAS numbers, but supplies an equally language-neutral “CUI” (Concept Unique Identifier).

### Normalizing indications

In the DID and its non-proprietary subset (Additional file 1), indications associated with each drug name are encoded at four basic levels of granularity.

- Raw entire value/string (column AQ): the raw source’s term/text, including entire DailyMed “Indications & Usage” sections converted to single-line sentences.
- Raw target/substring (column AR): a term/phrase within or based on the entire value/string, denoting a distinct indication concept. If the target/substring is the same as the entire value/string, it is flagged with “Y” in column AS.
- UMLS entry term (column AU) that best matches the target/substring *and* conforms to our semantic type preference for *phenotypes* (diseases and other biological conditions, processes, and functions; see below). UMLS mapping was done using ad hoc perl scripts designed to work with UMLS flat files (2013AA version), MetaMap [41], and/or NLM’s online UMLS browser [42]. Each UMLS entry term is tagged for whether it is preferred (“P”) or a non-preferred synonym (“S”) (column AX). For readability in the DID, all “P” terms were converted to proper case and all “S” terms were converted to lower case using Excel string functions.
- UMLS preferred term (column AV) and corresponding CUI (column AW) were computed from UMLS 2013AA flat files to unify all encoding at this level, even if raw values consisted of UMLS terms or CUIs (MeSH PA and NDFRT), except for mappings only available in more recent UMLS versions via NLM’s online browser.

### Indication semantic types

For UMLS encoding of indication concepts, we had a preference for UMLS concept terms classified under UMLS semantic types signifying *phenotypes* (diseases and other biological conditions, processes, and functions). The goal of this was to reduce encoding scatter. For example, the raw term “antibacterial agent” exactly matches a UMLS synonym under “Anti-Bacterial Agents” (CUI C0279516) classified under semantic type “Antibiotic” (A1.4.1.1.1.1). But calling a drug an “antibacterial agent” is equivalent to saying that its indication is “Bacterial Infections” (C0004623, classified under “Disease or Syndrome” B2.2.1.2.1). By mapping “Anti-Bacterial Agents”/C0279516 to “Bacterial Infections”/C0004623, raw data that encode to either are unified. This is tantamount to trading lexical match precision for increased terminological reduction (explained below).

In the DID and Additional file 1, initial indication mappings to non-phenotypic semantic type UMLS terms are encoded in columns BD-BL with their remapping to phenotypic type CUIs in AT-BC. If the initial non-phenotypic type mapping could not be mapped to a phenotypic type CUI, it is encoded in AT-BC. For example, “Cephalosporins” (a WHO-ATC category, among other instances) maps to C2266959/Antibiotic/A1.4.1.1.1.1, but is “stuck” there because UMLS had no phenotypic type term such as “cephalosporin activity”; “cephalosporin effect”; or “cephalosporin-sensitive infection.”

### Indication subtypes

In prior work [19, 20] we observed that drug indications are often classified or annotated by subtypes such as approved vs. non-approved, or treatment vs. prevention. The current work’s expanded raw data scope brought to light additional types with lexical cues such as therapeutic/pharmacologic class prefixes (“*Anti*diabetic”), suffixes (“Anxio*lytic*”), and head nouns (“beta-adrenergic *agonist*”; “Lipoprotein Lipase *Activators*”; “smoking cessation *adjunct*”). Some of these distinctions are likely to be even more substantial than treatment vs. prevention; e.g., “Antineoplastics” and “Carcinogens” both map to “cancer” but in opposite ways, one inhibitory or negative, the other causative or positive. This suggests an indication subtype hierarchy representing a gradient of granularity with raw terms like “treatment” and “prevention” at the bottom/leaf level and “negative” and “positive” at the top. In between would be lexical root forms such as “treat” representing “treats”; “treating”; “treatment”; etc. If so encoded in the DID, users could select the most appropriate indication subtypes and level of granularity for their use case. We identified indication subtypes based on Excel string searches (“treat”; “anti”; “inhibit”; etc.) in the raw entire value/string (column AQ).

### Terminological reduction

The inherent value of terminological normalization is the core principle of controlled vocabularies that have been used to organize, search, and represent information for over a century [43]. To measure the success of our terminological normalization efforts, we defined terminological reduction (TR) as TR = (*N* + X)/U, where *N* = number of unique normalized names, X = number of unique raw names which remain unnormalized, and U = number of unique original raw names.

## Results

### Database overview

The Merck in-house version of the DID (January 2015 release) contains 198,415 rows of data representing unique quadruplets of source, raw drug/chemical name, raw indication “target” term, and indication UMLS CUI. Across sources, there are 29,964 unique raw drug/chemical names, 10,938 raw indication target terms, and 192,008 unique raw drug/indication pairs. Additional file 1 is a copy of this spreadsheet minus 5,557 rows (3%) containing Merck proprietary information. Therefore reproducing these counts and the following analyses on Additional file 1 would yield slightly different quantitative results, but not substantially alter our qualitative conclusions. Additional file 1’s “schema” worksheet shows the DID schema and two example records.

### Drug name normalization

Drug name mapping to CAS numbers is encoded in DID columns E-H. CAS numbers were assigned to 87% of the DID rows and 71% of the unique raw drug names, providing TR of the unique names to 91%. The preferred authority ChemIDplus alone covered 84% of the rows and 68% of the unique raw drug names. Almost all (98%) of these CAS number mappings are based on exact (case-insensitive) matches to the ChemIDplus’ or other standard’s PT for that CAS number, or to a source-specified synonym (“<syn per source>”). The synonym matches were manually curated and obvious broader term (BT) and narrower term (NT) matches were reclassified as such. For BT and NT matches the directionality is raw-to-standard; e.g., raw “arformoterol fumarate” is a NT (a salt, derivative, analog, or formulation of) the closest ChemIDplus term which has a CAS number, “Arformoterol”. Also distinguished are quasi-synonym matches such as “cidofovir anhydrous”: “Cidofovir”. The intent is to offer users multiple match quality levels as options for filtering. The individual drug name mappings to ChEBI, ChemIDplus, and CTD are encoded in DID columns I-AC.

Drug name mapping to UMLS is encoded in DID columns AD-AM. UMLS CUI mapping, compared to CAS number mapping, produced superior coverage of DID rows (96% vs. 87%) and unique raw DB drug names (89% vs. 71%), and superior TR (85% vs 91%). The difference is at least partly due to the higher numbers of synonym and narrower UMLS matches, which may be an artefact of unequal curation effort or UMLS’ coverage of broad classes (e.g.,“antiseptics”) which by nature do not have CAS numbers.

### Indication normalization

Ninety-nine percent of DID rows represent unique triplets of raw data source (column B), drug name (column D), and indication target/substring (column AR), the other 1% representing compound matches where more than one UMLS term was needed to cover the indication concept completely. There are 10,938 unique values of the target/substring, of which 28 (0.3%) could not be mapped to UMLS. The rest mapped to 7,522 UMLS entry terms and thence to 6,227 UMLS PT/CUIs of the preferred semantic type (columns AT-BC), yielding a TR of 57%.

### Indication semantic type normalization

Unlike the drug name normalization mappings, the indication UMLS mappings have a sizable prevalence of quasi-synonym match types (column AT; 46% of rows, 30% of unique target/substrings). This is attributable to our preference for indication normalization to phenotypic-type UMLS terms, operationalized in the semantic type normalization step. Non-phenotypic-type terms were thus reduced from 29% of DID rows among initial UMLS mappings (columns BD-BL) to 3% among final (AT-BC), primarily terms of type “Pharmacologic Substance”/A1.4.1.1.1 (25% initial, 1% final). The prevalence rank of “Pharmacologic Substance”/A1.4.1.1.1 changed from first to 13th, reflecting the large contributions from ChEBI, CTD, MeSH, PDR, USAN, and WHO-ATC consisting or raw therapeutic/pharmacologic class terms (e.g., “Analgesics”; “Antineoplastics”; “Carcinogens”).

### Indication subtypes

Indication subtype data are contained in DID columns AN-AP. These data are very preliminary and incomplete. Supplementing and refining it is one of our ongoing extensions of this work.

### Comparison of sources

#### Coverage

Table 2 summarizes how much of the data was covered by each of the 12 sources after normalization. CTD covered by far the largest number of unique drug-indication relations (49%), followed by MeSH_PA, WHO_DD, and eVOC_ATC (10–14%), followed by the others (1–5%). With the exception of USAN_TC, this rank-order pattern also held for drug/chemical names alone. For indications alone, CTD also covered 49%, followed by DrugBank (34%), DailyMed (23%), USAN_TC (18%), NDFRT (16%), and the others (5–8%).

**Table 2:** Comparison of sources’ coverage of unique drug names, indication terms, and drug-indication relations after normalization

Source	%normalized		
	drug	indication	drug-indication pairs
CTD	33	49	49
MeSH_PA	27	6	14
WHO_DD	28	5	14
evoc_ATC	26	5	11
ChEBI	17	8	5
WHO_ATC	11	5	5
DrugBank	6	34	4
USAN_TC	23	18	4
NDFRT	6	16	3
DailyMed	4	23	2
evoc_eProj	3	6	1
PDR	3	5	1

Percentages are relative to total counts of 25,278 unique normalized drug names, 6,228 unique normalized indication terms, and 167,087 unique normalized drug-indication relations

#### Overlap

Table 3 summarizes overlap, a measure of the uniqueness of each source’s contribution to the DID, defined as the number of sources that contributed each unique drug and indication (target) term and drug-indication pair, before (raw) and after normalization, and the difference. Consistent with overall TR, the biggest effect of normalization was seen in the increase in shared indication terms with the descending rank-order following the tendency of each source to express indications in other-than-phenotypic-type terms (Table 3, column 9).

**Table 3:** Comparison of sources’ overlapping coverage of unique drug names, indication terms, and drug-indication relations before and after normalization

source	raw			normalized			change		
	drug	indic (target)	drug-indic pairs	drug	indic	drug-indic pairs	drug	indic	drug-indic pairs
All	1.64	1.30	1.02	1.87	1.80	1.14	0.23	0.50	0.12
evoc_ATC	1.62	3.40	1.03	2.09	6.77	1.38	0.47	3.37	0.35
WHO_ATC	3.81	3.37	1.07	4.66	6.67	1.96	0.85	3.30	0.89
WHO_DD	1.91	3.29	1.03	2.59	6.54	1.40	0.68	3.25	0.37
MeSH_PA	2.81	1.33	1.03	3.08	4.54	1.43	0.27	3.21	0.40
PDR	5.56	1.60	1.17	6.14	4.68	2.32	0.58	3.08	1.15
evoc_eProj	1.00	1.80	1.00	2.21	4.17	1.44	1.21	2.37	0.44
ChEBI	2.51	1.14	1.01	3.11	3.40	1.72	0.60	2.26	0.71
USAN_TC	3.03	1.47	1.02	3.34	3.30	1.81	0.31	1.83	0.79
DailyMed	4.68	1.60	1.15	5.18	2.79	1.48	0.50	1.19	0.33
NDFRT	5.46	2.45	1.41	6.24	3.61	1.76	0.78	1.16	0.35
DrugBank	5.63	1.49	1.20	6.24	2.62	1.70	0.61	1.13	0.50
CTD	2.41	1.53	1.03	2.62	2.06	1.08	0.21	0.53	0.05

Numbers represent the average number of sources sharing each term or term pair, computed within each source’s coverage. For example, the low outlier raw drug name score of 1.00 for evoc_eProj means that system only shares its raw drug names with itself, reflecting the use of Merck company codes as preferred terms in its internal data systems. When these are normalized, as much as possible, to public domain generic names, the score rises to 2.21; that is, these generic names representing evoc_eProj content are shared with enough other DID normalized content to push the non-self average from zero up to 1.21 (=2.21–1.00) even though some company codes do not yet have public domain generic names. Data are sorted in descending order of the change indication scores (column 9; ”change/indic”) for the individual sources

**Fig. 2:**
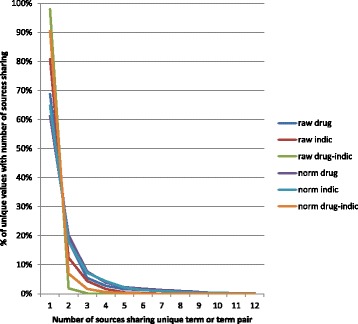
Zipf distributions of sources’ overlapping coverage of unique drug names, indication terms, and drug-indication relations before and after normalization

The pooled (all sources) shared term data can also be viewed as a Zipf distribution [44] (Fig. 2) showing, again, the larger effect of normalization on indication than drug terms or drug-indication pairs. Strikingly, no raw drug names were shared by more than 10 of our 12 resources, and only four normalized drug names were shared by all 12 (“Dexamethasone”; “Hydrocortisone”; “Methyldopa”; “Nitroglycerin”). The most-shared (by 11 sources) normalized drug-indication pairs were “Aspirin:Pain” and “Methyldopa:Hypertensive Disease” (the UMLS PT for “hypertension”).

#### Richness

Each source’s average numbers of indications per drug name and drug names per indication, before and after normalization, measure what might be called the “richness” of their drug-indication information. CTD had by far the highest (10) average raw indication targets per drug/chemical name, consistent with its low overlap and high coverage. Following CTD was a cluster in the range of 3.5–4 indication targets/drug that included DailyMed, MeSH_PA, DrugBank, and NDFRT, then a cluster in the 2.7–3.3 range that included WHO_DD, WHO_ATC, evoc_eProj, PDR, and evoc_ATC, and finally ChEBI (1.8) and USAN_TC (1.2). These numbers were little changed by normalization. The biggest changes were actually *negative* (0.4 *more* raw than normalized indications/drug for MeSH_PA and evoc_eProj).

The highest average numbers of drug names per raw indication target were provided by WHO_DD (69), evoc_ATC (56), and MeSH_PA (55). This same cluster also showed the biggest effect of normalization. At the low end, DailyMed and DrugBank data showed the most dramatic effect of processing, their average indications/drug increasing from approximately 1 (raw entire values) to 2 (raw targets) to 3 (normalized indications).

## Discussion

Our DID is intended to facilitate building practical, comprehensive, integrated drug ontologies. As for comprehensiveness, we achieved high source/data diversity as evidenced by a low overall degree of coverage overlap consistent with the idiosyncrasies of each source (non-drug chemicals, free text, hierarchical terms, etc.). Diversity is not equivalent to comprehensiveness, but is indicative of it. As for integration, indication normalization to phenotypic-type UMLS concepts provided substantial TR (57%). However, drug/chemical name normalization (TR 84%) was poor by comparison; therefore there was almost no effect of overall normalization on the average number of indications per drug.

WHO_DD’s, WHO_ATC’s, evoc_eProj’s, PDR’s, and evoc_ATC’s “richness” may be somewhat artificial in that it may be mainly due to WHO-ATC’s and PDR’s very general higher hierarchical categories. However, this feature may facilitate clustering of drug-indication relations and so explain WHO-ATC’s wide acceptance as a standard for drug classification and discovery research.

Because its true richness was not captured, DailyMed raises major issues for further development of the DID. These include the cost of dealing with the current (different) downloading, subsetting, and sectional parsing options, and developing better, less manual, free text-to-UMLS mapping methods. On the benefit side, methods applicable to DailyMed’s “Indications & Usage” sections are expected to be adaptable/re-usable for contraindications, side effects, and other dimensions of drug information. Relevance to clinical use cases is recognized [45] but DailyMed’s fit to early-stage drug discovery has been questioned [46]. NDFRT presents the opposite conundrum. In a spot check of two drugs, we [20] found major discrepancies between NDFRT’s *may_prevent* and *may_treat* relations and the approved clinical indications. Therefore these relations may be a poor fit to clinical drug ontology use cases. However, as a representation of *possible* drug indications conveyed by co-occurrence of MeSH terms in Medline, they may be ideal for early-stage drug discovery. Also, NDFRT’s *may_diagnose, has_mechanism_of_action,* and *has_physiological_effect* relations will be examined for future inclusion in the DID.

Finally, CTD’s high-coverage, low-overlap outlier status raises suspicion that its “marker/mechanism” subset (63%) may not be relevant to drug indications and therefore should be examined and possibly excluded from future DID releases.

## Conclusions

The DID is a database of structured drug-indication relations created using openly available, commercially available, and Merck proprietary information resources and terminological normalization tools. It is intended to facilitate building practical, comprehensive, integrated drug ontologies. The DID has good source/raw data diversity as measured by low coverage overlap, and significant integration/normalization as measured by terminological reduction. Numerous opportunities exist for data cleaning, addition, and other improvements. Our methodology could be adapted to the creation of other structured drug-disease databases such as for contraindications, precautions, warnings, and side effects.

## Additional file

Additional file 1: Drug-Indication Database non-proprietary subset. (XLSX 61806 kb)

## Abbreviations

AMA: American Medical Association
BAN: British Approved Name
BT: broader term
CAS number or CAS#: Chemical Abstracts Service Registry Number
ChEBI: Chemical Entities of Biological Interest
CTD: Comparative Toxicogenomics Database
CUI: Concept Unique Identifier [UMLS]
DB: database
DID: Drug-Indication Database
eVOC: electronic VOCabularies [Merck internal system]
FDA: U.S. Food and Drug Administration
GN: generic [drug] name
GO: Gene Ontology
InChI: International Chemical Identifier
MedDRA: Medical Dictionary for Reporting Activities
MeSH PA: Medical Subject Headings Pharmacological Action [relations]
MeSH: Medical Subject Headings
NDFRT: U.S. National Drug Formulary Reference Terminology
NLM: U.S. National Library of Medicine
NLP: natural language processing
NT: narrower term
OBO: Open Biological & Biomedical Ontologies
OTC: over-the-counter [drugs]
PDR: Physicians’ Desk Reference
PT: preferred term
SNOMEDCT: Systematized NOmenclature of MEDicine Clinical Terminology
TR: terminological reduction
UMLS: Unified Medical Language System
UNII: UNique Ingredient Identifier
USAN TC: United States Adopted Names Therapeutic Claim
USAN: United States Adopted Names
USP: United States Pharmacopeia
UTS: UMLS Terminology Services
WHO-ATC: World Health Organization Anatomic-Therapeutic-Chemical [classification]
WHO-DD: World Health Organization Drug Dictionary

## Footnotes

1 Following UMLS, we take “diseases” to be synonymous with “disorders.” We also mean “diseases” to convey the larger sense of pathological or aversive states that might otherwise be distinguished as signs, symptoms, abnormalities, deficiencies, injuries, etc.

2 Although different forms of the same generic name can in principle be specific to different indications, our conflation of NDFRT is not “lossy” because NDFRT appears to cross-generalize them regardless. For example, finasteride is marketed as a 1 mg tablet indicated to treat male-pattern baldness and a 5 mg tablet indicated to treat benign prostatic hyperplasia. But NDFRT has *may_treat* relations to both “Alopecia” and “Prostatic Hyperplasia” (the corresponding MeSH PTs) for all three: “Finasteride 1 mg Tab”; “Finasteride 5 mg Tab”; and “Finasteride.” In another example, “Bismuth” and all of its salt variants have relations to “Escherichia Coli Infections”; “Virus Diseases”; “Helicobacter Infections”; and “Dysentery, Bacillary” presumably related to bismuth subsalicylate’s gastrointestinal effects but definitely inappropriate for “Bismuth Hydroxide” which is a hazardous industrial chemical. In another example, radioactive and hazardous “Iodine, I-125” inappropriately shares the “Iodine” relations to “Burns”; “Leg Ulcer”; “Radiation Injuries”; “Staphylococcal Infections”; and “Surgical Wound Infection.”
